# AT1R gene rs389566 polymorphism contributes to MACCEs in hypertension patients

**DOI:** 10.1186/s12872-023-03223-w

**Published:** 2023-06-03

**Authors:** Jun-Yi Luo, Guo-Li Du, Yang-Min Hao, Fen Liu, Tong Zhang, Bin-Bin Fang, Xiao-Mei Li, Xiao-Ming Gao, Yi-Ning Yang

**Affiliations:** 1 State Key Laboratory of Pathogenesis, Prevention and Treatment of High Incidence Diseases in Central Asia, Urumqi, China; 2 Department of Cardiology, First Affiliated Hospital of Xinjiang Medical University, Urumqi, Xinjiang China; 3 Department of Endocrinology, First Affiliated Hospital of Xinjiang Medical University, Urumqi, Xinjiang China; 4 Xinjiang Key Laboratory of Medical Animal Model Research, Clinical Medical Research Institute of First Affiliated Hospital of Xinjiang Medical University, Urumqi, Xinjiang China; 5 People’s Hospital of Xinjiang Uygur Autonomous Region, 91 Tianchi Road, Urumqi, Xinjiang China

**Keywords:** Angiotensin II type 1 receptor (AT1R), Coronary artery disease (CAD), Hypertension, Major adverse cardiovascular and cerebrovascular events (MACCEs)

## Abstract

**Objective:**

To investigate the possible association between AT1R gene polymorphisms and major adverse cardiovascular and cerebrovascular events (MACCEs) in hypertension patients combined with or without coronary artery disease (CAD) in Xinjiang.

**Methods:**

374 CAD patients and 341 non-CAD individuals were enrolled as study participants and all of them have a hypertension diagnosis. AT1R gene polymorphisms were genotyped by SNPscan™ typing assays. During the follow-up in the clinic or by telephone interview, MACCEs were recorded. Kaplan–Meier curves and Cox survival analyses were used to explore the association between AT1R gene polymorphisms and the occurrence of MACCEs.

**Results:**

AT1R gene rs389566 was associated with MACCEs. The TT genotype of the AT1R gene rs389566 had a significantly higher probability of MACCEs than the AA + AT genotype (75.2% vs. 24.8%, P = 0.033). Older age (OR = 1.028, 95% CI: 1.009–1.0047, P = 0.003) and TT genotype of rs389566 (OR = 1.770, 95% CI: 1.148–2.729, P = 0.01) were risk factors of MACCEs. AT1R gene rs389566 TT genotype may be a predisposing factor for the occurrence of MACCEs in hypertensive patients.

**Conclusion:**

We should also pay more attention to the prevent of MACCEs in hypertension patients combined with CAD. Especially those elderly hypertensive patients carrying AT1R rs389566 TT genotype requires avoidance of unhealthy lifestyle, better management of blood pressure control and reduce the occurrence of MACCEs.

## Introduction

Coronary artery disease (CAD) and hypertensive are common diseases that endanger human health. As blood pressure regulatory system in the body, the renin-angiotensin system (RAS) is an important risk factors for CAD [1]. The angiotensin II (Ang II) type 1 receptor (AT1R) is involved in the classical physiological actions of Ang II, and plays a pivotal role in the pathogenesis of atherosclerosis in human [2].

Hypertension is a major risk factor for CAD and 25% of patients with CAD have hypertension [3, 4]. CAD is the first cause of morbidity and mortality in hypertension [5]. As referred above, AT1R is very important for the CAD, but its roles in pathogenesis of hypertension patients combined with CAD remains to be understood, although the associations between the AT1R polymorphisms, CAD and hypertension had been proved in French and English Caucasians population respectively [6, 7].

This study sequenced two single nucleotide polymorphisms (SNPs) of AT1R gene in all patients, and the differences in the distribution frequencies of these SNPs were compared between CAD patients and non-CAD patients combined with hypertension, and the association between AT1R gene polymorphisms and major adverse cardiovascular and cerebrovascular events (MACCEs) were analyzed.

## Materials and methods

### Study Population

In this case-control study, we recruited adult hypertension patients combined with CAD or non-CAD who were long-term residents of the Xinjiang region, China, and they were admitted to the Heart Center of the First Affiliated Hospital of the Xinjiang Medical University with symptoms of chest tightness or precordial discomfort during 2010–2018. Each subject signed an informed consent before participating in this study. Inclusion criteria: Guidelines for the diagnosis of hypertension [8]. Hypertension was defined as systolic blood pressure (SBP) above 140 mmHg or diastolic blood pressure (DBP) above 90 mmHg, having previously been diagnosed by a physician or taking antihypertensive drugs [9]. Exclusion criteria: patients with incomplete data and complicated with one or more than one disease, such as secondary hypertension, valvular heart disease, rheumatic heart disease, heart failure but no hypertension, congenital heart disease, systemic immune system diseases, and multiple organ failure.

### General data collection

The medical record system of our hospital was consulted according to the name and hospitalization certificate number, and the required data were collected according to the inclusion criteria, and data entry was performed using an Excel sheet. General data were collected including gender, age, body mass index (BMI), hypertension, type 2 diabetes mellitus (T2DM), Although people over the age of 50 used to smoke and drink, they have stopped smoking and drinking. Considering that smoking and drinking have caused damage to the vascular endothelium, this part of the population is still considered to have a history of smoking and drinking [10], family history of CAD, etc. Laboratory tests for blood glucose, lipids including cholesterol, triglycerides, high density lipoprotein cholesterol (HDL-c) and low-density lipoprotein cholesterol (LDL-c) were also collected.

### Diagnostic of MACCEs, CAD and Hypertension

MACCEs were considered as the endpoints, including cardiac and noncardiac death, nonfatal acute myocardial infarction, unplanned revascularization (new percutaneous coronary intervention or bypass cardiac surgery), malignant arrhythmia, development of congestive heart failure, and stroke. Cardiovascular events were defined according to the guidelines of the European Society of Cardiology (ESC) and the standardized definition of vascular and stroke endpoint events in the Clinical Data Interchange Standards Consortium (CDISC) clinical trial center [11, 12]. Typical symptom of CAD is exertional angina, with pressure pain in the precordial region during activity or emotional stress. It can radiate to the left shoulder or/and left upper arm for 5–10 min and can be relieved by rest or medications such as nitroglycerin. Diagnosis CAD is based on symptoms, signs and ancillary tests such as electrocardiography and coronary angiography (CAG). CAG is the gold standard for diagnosing CAD. Diagnosis of CAD should be at least one coronary arterial stenosis of 50% or its major branches in the CAG [13]. According to the Treatment of hypertension: The ESH/ESC guidelines recommendations [8], hypertension is diagnosed under the following conditions: systolic blood pressure (SBP) ≥ 140mmHg and / or diastolic blood pressure (DBP) ≥ 90mmHg on three different days in the absence of antihypertensive drugs; patients with a history of hypertension and currently taking antihypertensive drugs although their blood pressures were lower than 140 / 90mmHg.

### Genotyping assay

The same genotyping and unified standard biochemical tests were applied to all subjects. Two SNPs (rs389566 and rs1680760) of the AT1R gene whose minor allele frequencies (MAF) are more than 5% were selected from the HapMap human SNP database (www.hapmap.org). We also found that the above two SNPs were the tagging SNP of the Chinese Xinxiang hypertension population (MAF ≥ 5% and with R^2^ ≥ 0.8 as a cut-off in linkage disequilibrium pattern analysis) in the HapMap [14]. A total of 5 mL of fasting peripheral venous blood was drawn from the subjects into ethylenediaminetetraacetic acid (EDTA)-containing blood collection tubes, and plasma and blood cells were separated through centrifugation and stored in a − 80 °C refrigerator until further use. Plasma was were measured by biochemical indicator and blood cells were subjected to genomic DNA extraction using a whole blood genome extraction kit (Tiangen Biotech, China). AT1R gene polymorphism was detected by TaqMan® SNP genotyping qRT PCR. Genotyping accuracy was determined by genotypic concordance between replicate samples, and the accuracy of each SNP was 100%. The reaction system of qPCR amplification was composed of following reagents: 3 µL of TaqMan Universal Master Mix, 0.12 µL probes and 1.88 µL ddH_2_O in a 6 µL final reaction volume containing 50 ng DNA. Amplification cycling conditions were as follows: 95 °C for 5 min; 35 cycles of 95 °C for 15 s and 60 °C for 1 min.

### Statistical methods

SPSS 26.0 statistical software was used for statistical analysis. T-test was used for comparison between groups; χ chi-square test was used for comparison of count data. Cox regression was used for multi-factor analysis. The associations between patients’ survival rate and the AT1R gene polymorphism were evaluated using Kaplan–Meier analysis. A difference was considered statistically significant as P < 0.05 (two-sided).

## Results

### General clinical characteristics

In this study, we compared the general characteristics of patients between non-CAD and CAD patients combined with hypertension. We found that CAD patients tended to be older (55.7 ± 9.7 vs. 59.6 ± 10.7 years, P < 0.001), higher glucose levels of BMI (26.93 ± 3.85 vs. 26.07 ± 3.10 kg/m^2^, P = 0.030), SBP (132 ± 17 vs. 129 ± 18 mmHg, P = 0.023), DBP (80 ± 11 vs. 78 ± 12 mmHg, P = 0.043) compared with non-CAD patients. Patients with CAD also have higher levels of TC (4.17 ± 1.01 vs. 4.45 ± 1.18 mmol/L, P = 0.001), LDL-c (2.60 ± 0.83 vs. 2.76 ± 0.96 mmol/L, P = 0.010) and lower HDL-c (1.04 ± 0.29 vs. 0.99 ± 0.28 mmol/L, P = 0.020, Table 1).

**Table 1:** General characteristics in hypertension patients stratified with CAD

Characteristics	non-CAD (n = 341)	CAD (n = 374)	*P* Value
Age (years)	55.7 ± 9.67	59.6 ± 10.73	< 0.001
Gender, n (%)			
Male	173 (50.7%)	234 (62.6%)	0.001
Female	168 (49.3%)	140 (37.4%)	
Smoking, n (%)			
No	242 (71.0%)	229 (61.2%)	0.006
Yes	99 (29.0%)	145 (38.8%)	
Alcohol intake, n (%)			
No	251 (73.6%)	282 (79.5%)	0.582
Yes	90 (26.4%)	92 (20.5%)	
T2DM			
No	289 (84.8%)	261 (69.8%)	< 0.001
Yes	52 (15.2%)	113 (30.2%)	
SBP (mmHg)	132 ± 17	129 ± 19	0.023
DBP (mmHg)	80 ± 12	79 ± 13	0.043
BMI (kg/m^2^)	26.93 ± 3.85	26.07 ± 3.10	0.030
Glucose (mmol/L)	5.67 ± 1.96	8.44 ± 3.66	< 0.001
TG (mmol/L)	1.96 ± 1.48	2.13 ± 1.76	0.181
TC (mmol/L)	4.17 ± 1.01	4.45 ± 1.18	0.001
HDL–c (mmol/L)	1.04 ± 0.29	0.99 ± 0.28	0.020
LDL–c (mmol/L)	2.60 ± 0.83	2.76 ± 0.96	0.010

CAD: Coronary heart disease, BMI: Body mass index, SBP: Systolic blood pressure, DBP: Diastolic blood pressure, TG: Triglycerides, TC: Total Cholesterol, HDL-c: High Density lipoprotein cholesterol, LDL-c: Low density lipoprotein cholesterol, T2DM: Type 2 Diabetes Mellitus, P < 0.05 was considered significant difference

General characteristics and biochemical parameters between control (non-MACCEs) and MACCEs groups had been compared, as shown in Table 2. There was no significant difference regarding gender, smoking, alcohol intake, T2DM between these groups (P > 0.05). The prevalence of MACCEs in CAD patients was significantly higher than non-CAD patients (79.5% vs. 20.5%), P < 0.001. Patients with MACCEs showed higher blood glucose compared with those non-MACCEs patients (8.15 ± 3.42 vs. 6.93 ± 3.22 mmol/L), P < 0.001.

**Table 2:** **Biochemical parameters in hypertension patients stratified with MACCEs**

Biochemical parameters	non-MACCEs (n = 598)	MACCEs (n = 117)		*P* Value
Age (years)	57.1 ± 10.39	59.1 ± 9.79	< 0.001	
Gender, n (%)				
Male	337 (56.4%)	70 (59.8%)	0.488	
Female	261 (43.6%)	47 (40.2%)		
Smoking, n (%)				
No	391(65.4%)	80(68.4%)	0.533	
Yes	207(34.6%)	37(31.6%)		
Alcohol intake, n (%)				
No	440 (73.6%)	93 (79.5%)	0.180	
Yes	158 (26.4%)	24 (20.5%)		
T2DM				
No	461(77.1%)	89(76.1%)	0.810	
Yes	137(22.9%)	28(23.9%)		
CAD				
No	317 (53.0%)	24 (20.5%)	< 0.001	
Yes	281 (47.0%)	93 (79.5%)		
SBP (mmHg)	131 ± 18	129 ± 19	0.227	
DBP (mmHg)	80 ± 12	79 ± 13	0.412	
BMI (kg/m^2^)	26.55 ± 3.50	25.83 ± 3.06	0.097	
Glucose (mmol/L)	6.93 ± 3.22	8.15 ± 3.42	< 0.001	
TG (mmol/L)	2.04 ± 1.57	2.09 ± 1.94	0.810	
TC (mmol/L)	4.30 ± 1.10	4.40 ± 1.16	0.389	
HDL–c (mmol/L)	1.04 ± 0.29	0.99 ± 0.28	0.093	
LDL–c (mmol/L)	2.68 ± 0.89	2.73 ± 0.96	0.542	

MACCEs: Major adverse cardiovascular and cerebrovascular events, BMI: Body mass index, SBP: Systolic blood pressure, DBP: Diastolic blood pressure, TG: Triglycerides, TC: Total cholesterol, HDL-c: High density lipoprotein cholesterol, LDL-c: Low density lipoprotein cholesterol, T2DM: Type 2 Diabetes Mellitus, CAD: Coronary heart disease, P < 0.05 was considered significant difference

Patients with MACCEs showed higher age (Table 2) compared with those non-MACCEs patients (57.1 ± 10.3 vs. 59.1 ± 9.7 years), P < 0.001. We then compared characteristics among different age groups in Table 3. In 51–60 years old and over 60 years old groups, MACCEs occurrence increased significantly (31.6% and 54.7%, respectively, P = 0.001). The BMI, blood pressure, glucose, TG, HDL-c showed significantly difference among different age groups (P < 0.05). There was no difference regarding rs16860760, rs389566 TT genotype among different age groups (P = 0.932, P = 0.446 respectively).

**Table 3:** Comparison of general characteristics in patients with hypertension according to different ages

Characteristics	< 40 years old (n = 20)	41–50 years old (n = 178)	51–60 years old (n = 218)	> 60 years old (n = 293)	*P* Value
Gender, n (%)					
Male	18(90.0%)	139(77.7%)	123(56.2%)	127(42.8%)	< 0.001
Female	2(10.0%)	40(22.3%)	96(43.8%)	170(57.2%)	
Smoking, n (%)					
No	7(35.0%)	83(46.4%)	146(66.7%)	235(79.1%)	< 0.001
Yes	13(65.0%)	96(53.6%)	73(33.3%)	62(20.9%)	
Alcohol intake, n (%)					
No	9(1.7%)	104(19.5%)	164(30.8%)	256(48.0%)	< 0.001
Yes	11(55.0%)	75(41.9%)	55(25.1%)	41(13.8%)	
T2DM					
No	17(85.0%)	155(86.6%)	171(78.1%)	207(69.7%)	< 0.001
Yes	3(15.0%)	24(13.4%)	48(21.9%)	90(30.3%)	
CAD					
No	15(75.0%)	101(56.4%)	107(48.9%)	118(39.7%)	< 0.001
Yes	5(25.0%)	78(43.6%)	112(51.1%)	179(60.3%)	
SBP (mmHg)	137 ± 16	129 ± 17	129 ± 16	132 ± 20	0.039
DBP (mmHg)	87 ± 14	82 ± 12	80 ± 11	77 ± 13	< 0.001
BMI (kg/m^2^)	29.34 ± 3.52	27.03 ± 2.91	26.60 ± 3.61	25.63 ± 3.34	< 0.001
Glucose(mmol/L)	6.16 ± 2.06	7.06 ± 3.27	6.30 ± 2.63	7.76 ± 3.58	< 0.001
TG (mmol/L)	2.68 ± 3.04	2.36 ± 1.84	2.00 ± 1.73	1.84 ± 1.20	0.004
TC (mmol/L)	4.72 ± 1.17	4.37 ± 1.07	4.31 ± 1.15	4.26 ± 1.11	0.311
HDL–c (mmol/L)	1.02 ± 0.25	0.96 ± 0.23	1.04 ± 0.31	1.08 ± 0.30	0.001
LDL–c (mmol/L)	3.12 ± 0.79	2.74 ± 0.90	2.68 ± 0.87	2.62 ± 0.93	0.096
MACCEs, n (%)	1 (0.9%)	15 (12.8%)	37 (31.6%)	64 (54.7%)	0.001
RS389566 TT, n (%)	13 (2.8%)	116 (25.1%)	133 (28.7%)	201 (43.4%)	0.446
RS16860760 AA + AG, GG n (%)	20 (2.8%)	179 (25%)	219 (30.6%)	297 (41.5%)	0.932

BMI: Body mass index, SBP: Systolic blood pressure, DBP: Diastolic blood pressure, TG: Triglycerides, TC: Total Cholesterol, HDL-c: High density lipoprotein cholesterol, LDL-c: Low density lipoprotein cholesterol, MACCEs: major adverse cardiovascular events. P < 0.05 was considered significant difference

### Occurrence of MACCEs in patients with different genotypes of rs16860760 in AT1R gene

There was no significant difference regarding the frequency of MACCEs in different AT1R rs16860760 SNPs (P > 0.05), but the AT1R gene rs389566 polymorphism showed significant association with the probability of MACCEs in patients with hypertension (Table 4). And the patients carrying TT genotype at rs389566 locus had a higher risk of MACCEs than those carrying the AA + AT gene type (24.8% vs. 75.2%, P = 0.033).

**Table 4:** **AT1R gene polymorphisms in patients with MACCEs and control group**

Polymorphisms	non-MACCEs (n = 598)	MACCEs (n = 117)	*P* Value
*Rs16860760*			
AA + AG	62 (10.3%)	9 (7.7%)	0.599
GG	536 (89.6%)	108 (92.3%)	
*Rs389566*			
AA + AT	223 (37.3%)	29 (24.8%)	0.033
TT	375 (62.7%)	88 (75.2%)	

MACCEs: Major adverse cardiovascular and cerebrovascular events, P < 0.05 was considered significant difference

### Risk factors of MACCEs

**Fig. 1:**
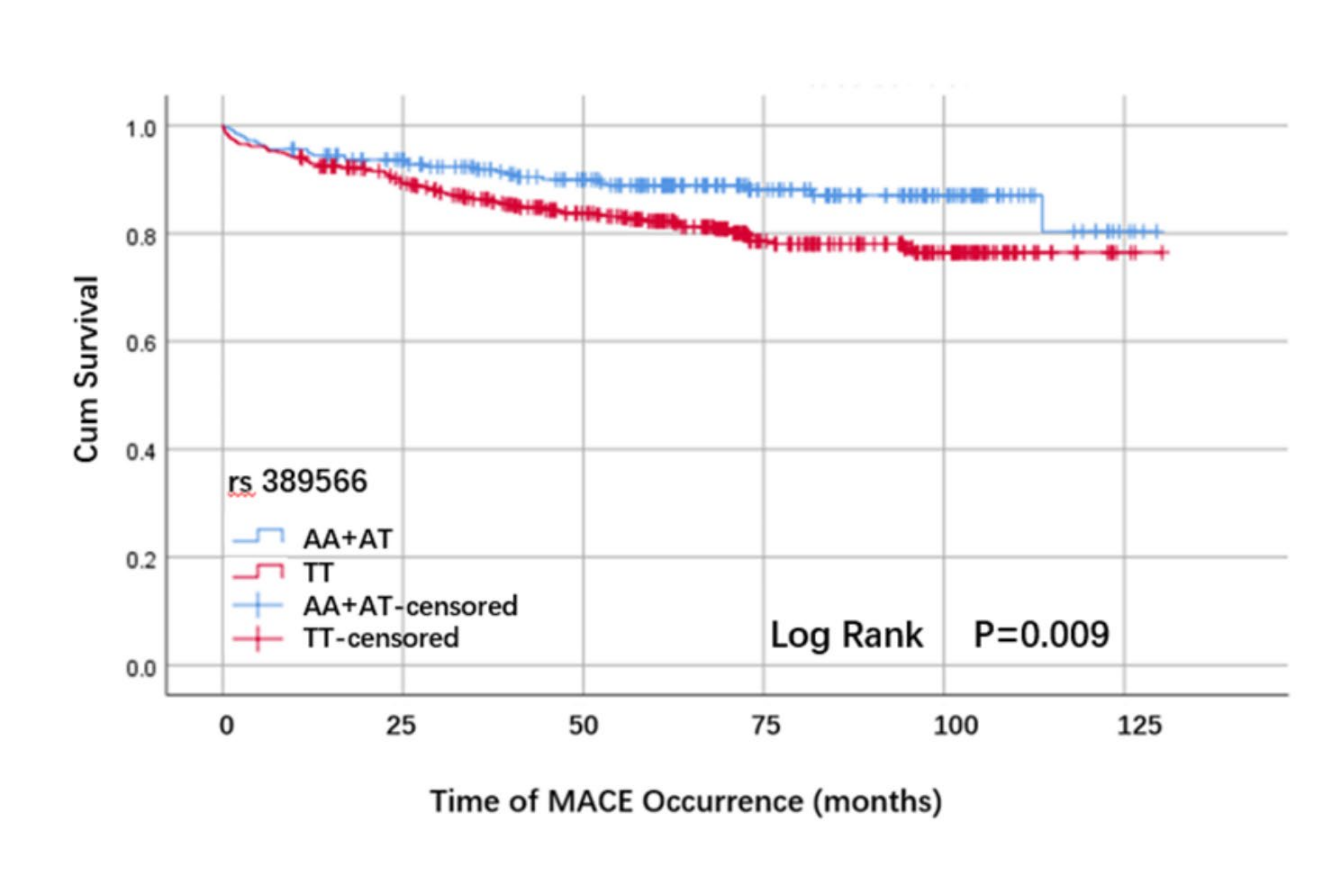
Kaplan-Meier curves of MACCEs survival analysis according to the rs389566 genotype

In the present study, the mean follow-up duration was 65.6 (38.3, 91.8) months. The Kaplan–Meier analysis revealed that the MACCEs-free cumulative survival rate in the TT genotype group was obviously lower than that in the AA + AT genotype group (P = 0.009, Fig. 1).

**Fig. 2:**
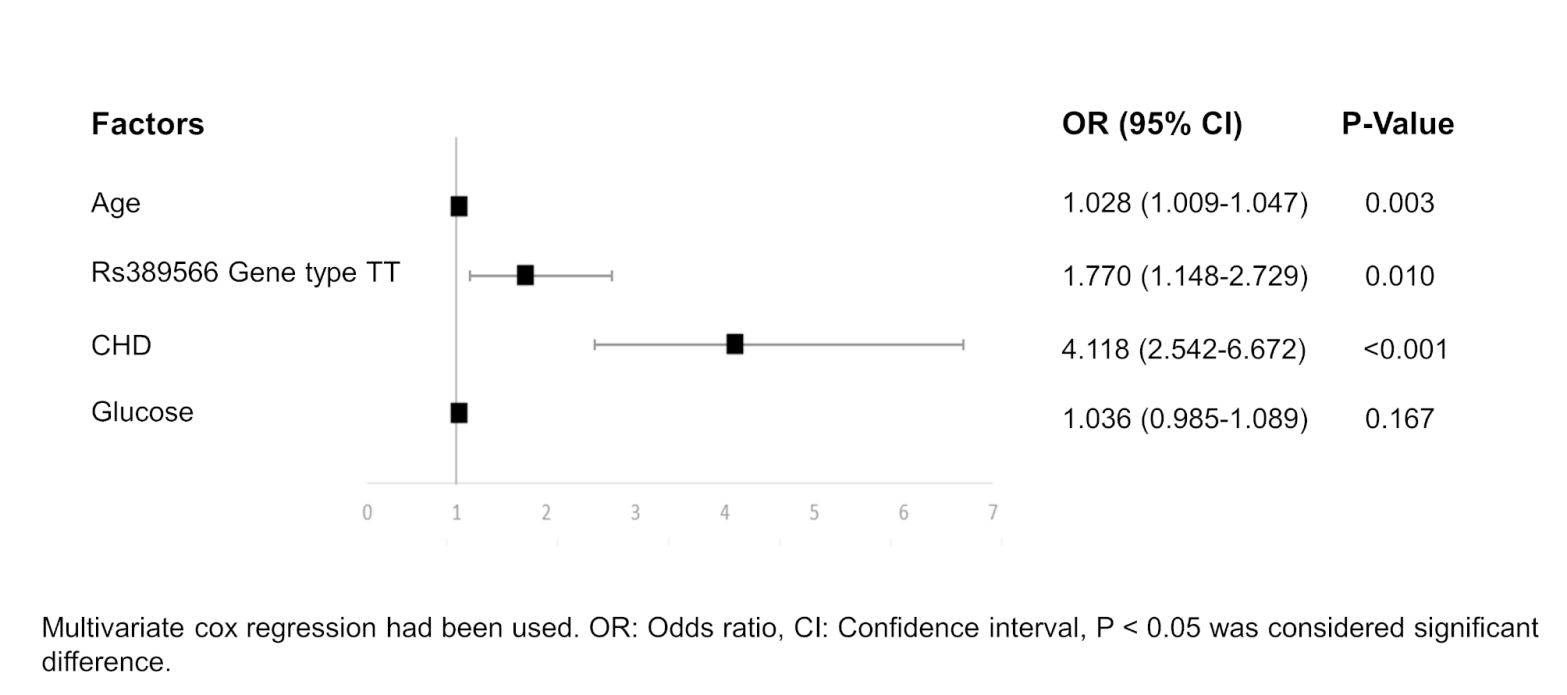
Cox regression analysis of the forest plot of MACCEs risk factors in hypertensive patients

Through univariate Cox survival analysis, we found that elderly, glucose, coronary artery disease, and rs389566 TT gene types may be risk factors for MACCEs in patients with hypertension. As shown in Table 5 and Fig. 2, age, AT1R gene rs389566 TT genotype, CAD and glucose variables were included to construct a multifactorial Cox proportional risk model. The results showed old age may be a predisposing factor on the occurrence of MACCEs (OR = 1.028, 95% CI: 1.009–1.047, P = 0.003), and rs389566 TT genotype may be a predisposing factor on the occurrence of MACCEs (OR = 1.770, 95%CI 1.148–2.729, P = 0.010).Patients with CAD were prone to MACCEs (OR = 4.118, 95%CI 2.542–6.672, P < 0.001). However, the glucose showed no significant different effect on occurrence of MACCEs in the final model (P > 0.05).

**Table 5:** Univariate and multivariate Cox analyses among the hypertension patients

Risk factors	Univariate cox regress			Multivariate cox regress	
	OR (95% CI) *P* Value			OR (95% CI) *P* Value	
rs389566 AA + AT/TT	1.731 (1.138–2.635)	0.010		1.770(1.148–2.729)	0.010
CAD	4.912 (3.128–7.714)	< 0.001		4.118(2.542–6.672)	< 0.001
Age	1.041(1.023–1.060)	< 0.001		1.028(1.009–1.047)	0.003
Gender	0.789 (0.545–1.142)	0.208		-	-
BMI	0.951(0.890–1.016)	0.136		-	-
SBP	0.992 (0.982–1.002)	0.135		-	-
DBP	0.992(0.977–1.007)	0.278		-	-
Glucose	1.107(1.060–1.156)	< 0.001		1.036(0.985–1.089)	0.167
TG	1.008(0.897–1.132)	0.895		-	-
TC	1.101(0.930–1.303)	0.264		-	-
HDL-c	0.578(0.297–1.127)	0.108		-	-
LDL-c	1.108(0.900-1.363)	0.334		-	-

BMI: Body mass index, SBP: Systolic blood pressure, DBP: Diastolic blood pressure, TG: Triglycerides, TC: Total Cholesterol,

HDL-c: High density lipoprotein cholesterol, LDL-c: Low density lipoprotein cholesterol, MACCEs: major adverse cardiovascular

events. CAD: Coronary heart disease, OR: Odds ratio, CI: Confidence Interval, P < 0.05 was considered significant difference

## Discussion

Many factors influence the occurrence of MACCEs, such as family history of CAD, smoking, obesity, hypertension, diabetes, abnormal lipid metabolism, insulin resistance, and homocysteine mia [15]. In the present study, AT1R gene rs389566 TT genotype was found to be associated with the occurrence of MACCEs in hypertension patients.

Cardiovascular disease is the leading cause of death worldwide [16], and hypertension is the most common chronic disease and the most important risk factor for cardiovascular disease [17]. Although CAD mortality rates have gradually declined in Western countries over the past few decades, the condition still causes about one-third of deaths in people over 35 years of age [18]. MACCEs remain the major cause of mortality and morbidity in patients both in hypertension or CAD patients [19, 20].

However, it has been reported in the literature that the incidence of MACCEs is significantly higher in CAD combined with hypertension patients compared with non-CAD or non-hypertension patients [21, 22], but the reasons remain to be unknown. The traditional risk factors of MACCEs include fasting glucose, heart rate variability, blood pressure [23–26] and dyslipidemia [27]. As previous reported, AT1R gene polymorphism was found to be associated with the development of CAD in Chinese population [28, 29]. Here we found AT1R rs389566 TT genotype may be an independent risk factor for the development of MACCEs in patients with hypertension especially those combined with CAD. The main effects of Renin-Angiotensin-Aldosterone System (RAAS) on cardiovascular system are atherosclerosis and hypertension, leading to congestive heart failure and MACCEs [30]. And Ang II also promotes the development of atherosclerosis through AT1 receptors, stimulating the secretion of inflammatory mediators, and converting stable plaques into vulnerable plaques [31]. Overexpression of the AT1R gene leads to myocardial hypertrophy and ventricular remodeling [32]. The previously study demonstrates that the AT1R polymorphism is associated with abnormal coronary vasoconstriction which causes rupture of plaque and thrombus formation [33].

Xinjiang is a multi-ethnic region, and there are 13 ethnic groups living here. Most residents like high-salt, high-calorie high-fat diet and low vegetable intake. Influential factors of unhealthy living habits lead to high incidence of obesity, hyperglycemia, hyperlipidemia and hypertension. These risk factors increase the risk of cardiovascular and cerebrovascular diseases in hypertensive patients [34]. According to the best knowledge of ours, we have not found the prevalence of MACCEs in Xinjiang population, but other studies had shown that valvular heart diseases were more common in the Han and Kazakh compared with the Uyghur [35]. The prevalence of chronic heart failure was higher in the Kazakh than the Han or Uyghur [36], and the prevalence of peripheral arterial disease was higher in the Uyghur than the Kazakh [37]. Our study found that AT1R gene mutation was associated with the occurrence of MACCEs in hypertension patients in the Xinjiang. The patients with hypertension carrying TT genotype of the AT1R gene rs389566 were prone to MACCEs. Previous studies have been conducted on AT1R gene polymorphisms in the Chinese population, but mainly on hypertension, atherosclerosis, cardiovascular disease risk factors, and intravascular restenosis. The association of AT1R gene polymorphisms with the occurrence of MACCEs events has not been reported before. Most previous studies have focused on the association of the AT1R rs5186 (A1166C) locus polymorphism and acute myocardial infarction in Caucasian, Asian, African, Brazilian, and Durban populations, and the C allele was proved to be a risk factor for occurrence of myocardial infarction [38]. In Asia, previous studies [38, 39] reported that AT1R A1166C polymorphism may influence the occurrence of myocardial infarction susceptibility in Chinese. However, the sample size of these studies is relatively small, and fewer studies have focused on the relationship between AT1R rs16860760 and MACCEs. In the present study, we found the significant association between AT1R rs389566 polymorphism and MACCEs in Chinese hypertensive population which could help provide a clinical basis for future targeted interventions.

Different ethnic groups in Xinjiang may have different genetic backgrounds, diets and living environments, and it is necessary to carry out further genetic and laboratory investigations to distinguish the differences among different ethnic groups.

Besides AT1R gene polymorphism, the age is also a factor affecting the occurrence of MACCEs. Our study found that the occurrence of MACCEs is higher in older age population, Patients with hypertension over 60 years are more likely to occur MACCEs and the prevalence is about 54.7% and it was consistent with previous study [40]. For aged population, MACCEs prevention should be emphasized in future.

Our study confirmed that AT1R rs389566 TT genotype increased the occurrence of MACCEs in hypertension patients.

## Conclusion

In summary, this study provides the current status of risk factors for the occurrence of MACCEs in hypertensive patients combined with CAD in Xinjiang, China. Angiotensin receptor blocker (ARB) is an important drug for the treatment of hypertension and heart failure. We found that the incidence of MACCEs in hypertensive patients with TT genotype of AT1R rs389566 gene polymorphism increased after gene mutation. so, the regulation of AT1R is very important for hypertension patients. ARB drugs can act on AT1R. In theory, the standardized application of ARB drugs can reduce the occurrence of MACCEs in hypertension patients. The incidence of MACCEs in hypertension patients combined with CAD is higher. We should actively prevent and treat CAD to prevent MACCEs. The present findings provide potential intervention targets for the prognosis of patients who are at high risk of MACCEs, and this will help clinician do genomics-based personalized therapy in future.

## Limitation

This study also has some limitations. First, the sample size was not large enough. Second, participants in the current study were recruited only at the First Affiliated Hospital of Xinjiang Medical University, which may not necessarily reflect the true prevalence of hypertension combined with CAD and the occurrence of MACCEs at the provincial or national level. Thirdly, in our study, MACCEs is the end point. Of the 715 subjects we included, 117 cases (23%) had MACCEs and the incidence of MACCEs was low. In order to reflect the universality of MACCEs with AT1R rs389566 genotype in hypertensive population, it needs longer follow-up and larger sample size support. Finally, we focused our interest on the AT1R gene polymorphism: as discussed, many other factors are involved in MACCEs and may cause increase in occurrence. Broader analyses are therefore encouraged to better understand the complexity of the MACCEs occurrence process.

## Data Availability

The datasets used and analyzed during the current study available from the corresponding author on reasonable request.
