# Correction to: AT1R gene rs389566 polymorphism contributes to MACCEs in hypertension patients

**DOI:** 10.1186/s12872-023-03368-8

**Published:** 2023-06-30

**Authors:** Jun-Yi Luo, Guo-Li Du, Yang-Min Hao, Fen Liu, Tong Zhang, Bin-Bin Fang, Xiao-Mei Li, Xiao-Ming Gao, Yi-Ning Yang

**Affiliations:** 1 State Key Laboratory of Pathogenesis, Prevention and Treatment of High Incidence Diseases in Central Asia, Urumqi, China; 2 Department of Cardiology, First Affiliated Hospital of Xinjiang Medical University, Urumqi, Xinjiang, China; 3 Department of Endocrinology, First Affiliated Hospital of Xinjiang Medical University, Urumqi, Xinjiang, China; 4 Xinjiang Key Laboratory of Medical Animal Model Research, Clinical Medical Research Institute of First Affiliated Hospital of Xinjiang Medical University, Urumqi, Xinjiang, China; 5 People’s Hospital of Xinjiang Uygur Autonomous Region, 91 Tianchi Road, Urumqi, Xinjiang, China

Following publication of the original article [1], In this article the statement in the Funding information section was incorrectly given as *“The survey was funded by the Xinjiang Young Scientific and Technical Talents Training Project (2020Q087, 2019Q040), the National Natural Science Foundation of China (82160054, 81960078), and State Key Laboratory of Pathogenesis, Prevention and Treatment of High Incidence Diseases in Central Asia, Xinjiang Medical University (SKL-HIDCA-2021-XXG4).”* and the statement should read as *“The survey was funded by the Xinjiang Young Scientific and Technical Talents Training Project (2020Q087, 2019Q040), the National Natural Science Foundation of China (82160054, 81960078, 81960073), and State Key Laboratory of Pathogenesis, Prevention and Treatment of High Incidence Diseases in Central Asia, Xinjiang Medical University (SKL-HIDCA-2021-XXG4).”*

The original article has been corrected.
